# Acute Inflammatory Demyelinating Polyneuropathy and a Unilateral Babinski/Plantar Reflex

**DOI:** 10.1155/2008/134958

**Published:** 2007-11-12

**Authors:** Davide Cattano, Brian O'connor, Ra'ad Shakir, Francesco Giunta, Mark Palazzo

**Affiliations:** ^1^Department of Surgery, University of Pisa, 56126 Pisa, Italy; ^2^Department of Anesthesiology, Washington University School of Medicine, Saint Louis, MO 63110, USA; ^3^Kings College Hospital, London SE5 9RS, UK; ^4^Charing Cross Hospital, Hammersmith Hospitals NHS Trust, London W6 8RF, UK

## Abstract

Acquired acute demyelinating peripheral polyneuropathy (AADP) is a general classification of pathologies that could affect secondary the peripheral nervous system. They are characterized by an autoimmune process directed towards myelin. Clinically they are characterized by progressive weakness and mild sensory changes. Acute inflammatory demyelinating polyneuropathy often is referred to as Guillain-Barré syndrome (GBS). GBS is the major cause of acute nontraumatic paralysis in healthy people and it is caused by autoimmune response to viral agents (influenza, coxsackie, Epstein-Barr virus, or cytomegalovirus) or bacterial infective organisms (Campylobacter jejuni, Mycoplasma pneumoniae). A detailed history, with symptoms of progressive usually bilateral weakness, hyporeflexia, with a typical demyelinating EMG pattern supports the diagnosis. Progressive affection of respiratory muscles and autonomic instability coupled with a protracted and unpredictable recovery normally results in the need for ICU management. We present a case report of a patient with a typical GBS presentation but with a unilateral upgoing plantar reflex (Babinski sign). A unifying diagnosis was made and based on a literature search in Pubmed appears to be the first described case of its kind.

A 66-year-old non-insulin-dependent diabetic man receiving metformin was admitted to hospital following a week of fever, dyspnoea, and left-sided chest and left-arm pains. Past medical history of note was hip replacement 6 months prior to admission and a presumed diagnosis of nerve root compression causing sciatica. The patient revealed that three weeks before admission, he saw his general practitioner feeling feverish and generally weak and an initial course of ampicillin and erythromycin failed to prevent progressive dyspnoea which prompted referral to hospital under the care of a chest physician.

On admission, positive findings included tachypnoea (40 breaths/minute) with pulse oximetry saturations of 99% on 2 litres/min nasal oxygen and sinus tachycardia (150 beats/minute) with blood pressure (BP) of 140/75 mmHg. He had global muscle weakness, worse in his legs, and associated upper- and lower-limb areflexias. Unexpectedly, he also had a right Babinski/plantar reflex. The latter was considered to be possibly related to cervical cord pathology in the absence of obvious central neurological changes. There were no sensory or cranial nerve deficits.

Arterial blood gases revealed respiratory alkalosis: pH 7.5, PaCO_2_ 3.22 Kpa, PaO_2_ 13.5 Kpa, HCO_3_ 18.9 mmol/L, and base deficit − 4 mmol/L. Abnormal results included white blood count of 8.9 × 10^9^ cells/L (91% neutrophilia), platelet count of 27 × 10^9^ cells/L, C reactive protein (CRP) of 333 mg/L (normal range is 0–7 mg/L), alkaline phosphatase of 173 U/L (30–95 U/L), alanine aminotransferase of 118 U/L (8–45 U/L), aspartate aminotransferase of 225 U/L (10–35 U/L), *γ*-glutamyl transferase of 159 U/L (5–50 U/L), and calcium of 2.08 mmol/L (2.2–2.6 mmol/L). Urine analysis showed protein and blood.

Six hours after admission, the patient became hypotensive (BP of 80/40 mmHg), peripherally vasoconstricted, and oliguric and he was admitted to the intensive care unit. Initial blood culture results revealed Gram-positive cocci and vancomycin treatment was initiated pending sensitivities. Supportive management included rapid fluid resuscitation (5000 mL), noradrenaline infusion titrated to restore systolic blood pressure to premorbid values once volume is repleted, and furosemide infusion (4 mg/h) was started to promote urine output. Day-two blood culture results revealed a methicillin-sensitive *Staphylococcus aureus,* and vancomycin was continued.

Nerve conduction studies on day 3 showed globally absent sensory responses in upper and lower limbs, absent lower-limb compound muscle action potentials (CMAPs), and reduced upper-limb median CMAP amplitudes with evidence of conduction block. Right median nerve motor conduction was reduced to 32 m/s and F-wave latencies were absent in median and common peroneal nerves. These findings together with clinical pictures were suggestive of an acute inflammatory demyelinating polyneuropathy (AIDP). Further investigations for ganglioside GM1, antiacetylcholine receptor, *Campylobacter jejuni*, atypical pneumonia, and viral antibodies were noncontributory. Creatine kinase concentrations were normal. Spinal and pelvic X-rays and abdominal ultrasound were normal. A five-day course of immunoglobulin (0.4 mg/kg) was commenced on day 3.

Although blood concentration-controlled vancomycin therapy targeted to 20 mg/L and the start of immunoglobulin treatment, the patient's condition gradually deteriorated by day 4 with the development of type 2 respiratory failure which required ventilation. *S. aureus* continued to be isolated from blood cultures and fusidic acid treatment was added to provide synergy and better tissue penetration. With supportive measures and immunoglobulin therapy, the patient made progress so that by day 8 renal and respiratory functions and limb strength had improved sufficiently for the patient to be weaned from ventilation. However, worryingly, the acute-phase markers of inflammation (CRP, fibrinogen, and white cell count) continued to rise. On day 9, the patient developed mild dysarthria. Clinical examination revealed for the first time a midsystolic murmur at the left sternal edge. Transthoracic echocardiogram revealed mitral valve regurgitation and a large vegetation on the posterior leaflet. In view of these findings, therapy was changed to flucloxacillin and gentamicin. On day 10, CT head scan showed a small hemorrhagic infarct in the left parietooccipital area with a similar mild focus in the left temporal grey matter which was thought to account for the dysarthria and Babinski response. On further discussion with the patient's wife, it transpired that the patient had extensive dental surgery 3 months prior to admission. On day 11, the patient had an urgent mitral valve replacement.

Repeating EMG investigations on day 27 showed a dramatic improvement with evident sensory potentials, median nerve conduction velocities within the normal range, and obtainable evoked muscle potentials. F-wave latencies, previously absent, were detectable but slightly delayed.

The patient was discharged from hospital 6 weeks after admission with minimal neurological deficit.

## 1. DISCUSSION

This diabetic patient initially presented with a history of general weakness and a fever which on initial examination and results of investigations fitted in with a diagnosis of *S. aureus* septic shock of uncertain origin complicated by EMG-proven AIDP. Unusually, the patient also repeatedly had a clinically elicited unilateral Babinski response with no other upper motor neuron signs or central disturbance. In view of the previous history of back pain and absent central symptoms, this was thought to be possibly related to a coincidental cervical lesion. However, initial spinal radiology was normal.

However, it was the sudden development of dysarthria which led to a unifying diagnosis of acute bacterial endocarditis unusually complicated by acute inflammatory demyelinating polyneuropathy and cerebral emboli. This produced clinical signs of upper and lower motor neuron lesions.

The association of bacterial endocarditis with both AIDP and cerebral embolic infarcts has not been previously described. Acute bacterial endocarditis is complicated by neurological changes in 25–40% of cases [1–4]. The most common neurological complications are central causing upper motor neuron changes and they are most frequently represented by cerebral embolism, meningitis, micrabscess, and mycotic aneurysm with or without subsequent haemorrhage. Neurological complications seem to be more frequent when *S. aureus* is the infecting organism [1]. Global peripheral nervous involvement is much less common and if peripheral nervous changes are observed, it is usually an acute mononeuropathy [4]. There have been however reports of axonal polyneuropathy or critical illness polyneuropathy associated with acute bacterial endocarditis, but these are usually a specific manifestation of established multiple organ failure [5–7].

Our patient presented with clinical and EMG findings typical of AIDP, which can be associated with any number of infections, but a rare manifestation of acute bacterial endocarditis. It was sufficiently severe to result in progressive type 2 respiratory failure, but it responded well to a course of immunoglobulin such that he was weaned from ventilation in a few days.

The unilateral Babinski response was difficult to account for in the absence of evidence of central or high spinal lesions. AIDP typically affects peripheral and cranial nerves but not the CNS [8] and therefore it is unlikely to be the cause of our patient's unilateral Babinski response.

This report illustrates that when an unusual constellation of clinical signs is observed, a unifying diagnosis should always be sought. In this case, the rarer global acute demyelinating lower motor neuron changes dominated, mostly disguised the upper motor neuron signs of hyperreflexia, and increased tone usually associated with a Babinski response.

The second lesson from this case is that history taking is usually better once a diagnosis is made. Suspicion would have been heightened and cardiac investigations were performed earlier with a directed history for a patient with pyrexia of unknown origin. Fortunately, our patient received early mitral valve replacement and was discharged home.
